# P-1337. Analyzing SARS-CoV-2 Case Numbers and Clustering to Predict a Nursing Home Outbreak

**DOI:** 10.1093/ofid/ofae631.1514

**Published:** 2025-01-29

**Authors:** Yasin Abul, Kevin McConeghy, Frank Devone, Christopher Halladay, Stefan Gravenstein, James Rudolph

**Affiliations:** Brown University, Providence, Rhode Island; COIN-LTSS, Providence Veterans Affairs Medical Center, Providence, Rhode Island; Center of Innovation in Long Term Services and Supports, Providence VA Medical Center, Providence, Rhode Island; Providence VAMC, Providence, Rhode Island; Brown University, Providence, Rhode Island; Center of Innovation in Long Term Services and Supports, Providence VA Medical Center, Providence, Rhode Island

## Abstract

**Background:**

Severe Acute Respiratory Syndrome Coronavirus 2 (SARS-CoV-2), can spread rapidly in nursing homes (NH). Intervening to reduce spread should follow a signal predicting an imminent "outbreak". Various thresholds for declaring a COVID-19 outbreak have considered local factors, viral variants, case identification timelines and vaccination rates. We evaluated the number and interval between initial SARS-CoV-2 infections (cases) most predictive of future cases using VA Community Living Centers (CLCs) as NH analogues.

**Methods:**

We captured SARS-CoV-2 PCR test results from individual NH neighborhoods (specialized wards). After an initial index case, we evaluated for additional cases over the next 28 days by R² analysis and 14 days by Classification and Regression Trees (CART) analysis. We evaluated the R^2^ goodness-of-fit measure from separate linear models by neighborhood to measure the predictive power of various outbreak definitions, adjusted for the number of residents in the neighborhood. Also, we used CART creating a tree-like structure to predict continuous target variables to see the effect of census and time at only 14 days.

**Results:**

We included 136 CLCs housing 16,339 Veterans, mean age of 74, mostly male (96%) and white (68%), in 490 neighborhoods, representing 1,252,037 Veteran exposure days from 12/18/2021 to 06/18/2022, and 1,765 SARS-CoV-2 infections. We divided neighborhoods by small [< 10 Veterans (n=30,390)], medium [10-15 veterans (n=25,349)], and large [ >15 veterans (n=31,899)]. The most predictive outbreak definition for future COVID-19 cases over the next 14 days for our linear model analysis was: 2 cases across all neighborhoods, 4 cases for large, 2 for medium and 1 for small size neighborhoods. Our preliminary CART analysis suggested the first case was predictive of another case in 5 days, but if ≥4 occurred in 5 consecutive days, it predicted a large-scale outbreak with 10 or more cases over the next 14 days if the neighborhood is large enough to have that many cases.

**Conclusion:**

Our analysis suggests when 4 SARS-CoV-2 cases cluster in five days in a large neighborhood, a large outbreak is imminent, potentially warranting aggressive control measures. However further refinement of our methods is needed to confirm this conclusion.

**Disclosures:**

**Yasin Abul, MD**, Moderna: Grant/Research Support|Moderna, Abt, CDC: Grant/Research Support **Kevin McConeghy, Pharm.D.**, Genentech: Grant/Research Support|GlaxoSmithKline: Grant/Research Support|Moderna: Grant/Research Support|Sanofi-Pasteur: Grant/Research Support|Seqirus: Grant/Research Support **Stefan Gravenstein, MD, MPH**, CDC: Advisor/Consultant|CDC: Grant/Research Support|Genentech: Advisor/Consultant|Genentech: Grant/Research Support|Genentech: Honoraria|GlaxoSmithKline: Advisor/Consultant|GlaxoSmithKline: Grant/Research Support|GlaxoSmithKline: Honoraria|Janssen: Advisor/Consultant|Janssen: Grant/Research Support|Janssen: Honoraria|Moderna: Advisor/Consultant|Moderna: Grant/Research Support|Moderna: Honoraria|NIH: Grant/Research Support|Pfizer: Advisor/Consultant|Pfizer: Grant/Research Support|Pfizer: Honoraria|Sanofi: Advisor/Consultant|Sanofi: Grant/Research Support|Sanofi: Honoraria|Seqirus: Advisor/Consultant

